# Evaluation of progression of chronic kidney disease in dogs with myxomatous mitral valve disease

**DOI:** 10.3389/fvets.2023.1200653

**Published:** 2023-08-24

**Authors:** Hyejin Yun, Yoonhoi Koo, Taesik Yun, Yeon Chae, Dohee Lee, Sijin Cha, Jeeyune Kim, Hakhyun Kim, Mhan Pyo Yang, Byeong Teck Kang

**Affiliations:** ^1^Laboratory of Veterinary Internal Medicine, College of Veterinary Medicine, Chungbuk National University, Cheongju, Chungbuk, South Korea; ^2^College of Veterinary Medicine, Kyungpook National University, Daegu, South Korea

**Keywords:** cardiovascular-renal disease, chronic kidney disease, myxomatous mitral valve disease, heart, kidney

## Abstract

**Introduction:**

Cardiovascular and renal diseases are known to affect each other in the cardiovascular renal axis disorder (CvRD). Although CvRD, which includes myxomatous mitral valve disease (MMVD) and chronic kidney disease (CKD), has been described in dogs, there are only a few reports on the progression of CKD in accordance with the severity of MMVD. The aim of this study was to evaluate whether the presence of MMVD is associated with the rate of progression of CKD in dogs. The time from the initial diagnosis to the worsening of the International Renal Interest Society (IRIS) stage and the time for the occurrence of hyperphosphatemia and isosthenuria were evaluated.

**Materials and methods:**

In this retrospective study, CKD progression was determined as an increase in the IRIS stage by at least one level and the development of hyperphosphatemia or isosthenuria. The CKD progression was compared in dogs with and without comorbid MMVD.

**Results:**

Dogs with CKD were divided into two groups: dogs with and without MMVD (*n* = 63, concurrent group; *n* = 52, CKD group, respectively). The concurrent group was further divided into two subgroups based on the American College of Veterinary Internal Medicine guidelines (B1 group, *n* = 24; B2 group, *n* = 39). The time for progression of CKD from IRIS stage 1 to IRIS stage 2 was significantly shorter in the concurrent group than in the CKD group (log-rank test, *p* < 0.001). MMVD was associated with an increased risk of progression from stage 1 to stage 2 (hazard ratio, 6.442; 95% confidence interval (CI), 2.354 to 18.850; *p* < 0.001). The timing of the onset of hyperphosphatemia or isosthenuria in the concurrent group and the CKD group was not significantly different.

**Conclusion:**

The results of this study suggest that MMVD could be a risk factor for the progression of CKD. Our findings may help predict the prognosis of dogs with both CKD and MMVD compared to CKD only.

## 1. Introduction

The kidneys and heart have a shared physiology, as these organs regulate vasomotor tone and fluid balance (1). Consequently, dysfunction or disease in either of these organ systems can initiate and perpetuate dysfunction or disease in the other system mediated through complex neurohormonal feedback mechanisms (2). This combined dysfunction of the heart and kidney is defined as cardiorenal syndrome (CRS) in human medicine and cardiovascular renal axis disorder (CvRD) in veterinary medicine (3). CvRD is a veterinary medical term that is defined as a syndrome in which renal and heart diseases affect each other, causing simultaneous progression of both; it is classified into five subtypes (3). The types of renal dysfunction due to impairment of cardiac function are classified into unstable CvRD_H_, which is caused by acute heart disease, and stable CvRD_H_, which is caused by chronic disease. The types of cardiac dysfunction due to renal dysfunction are classified as unstable CvRD_K_, which is caused by acute primary worsening of the kidney, and stable CvRD_K_, which is caused by primary renal disease. Additionally, CvRD_O_ is defined as cardiac and renal impairment secondary to a chronic or acute systemic condition.

A study by Levin et al. (4) reported that out of 244 human patients who were newly diagnosed with heart disease or had an existing heart disease, 47 patients had CRS (4). Owing to an increased interest in identifying the relationship between these two organ systems, several recent human patient-based studies have investigated the risk of chronic kidney disease (CKD) progression in individuals with cardiovascular disease and concluded that cardiovascular disease is a risk factor for CKD progression (4–6). Potential pathophysiological mechanisms include decreased renal perfusion due to low cardiac output, venous congestion, neurohormonal activation, oxidative stress, and cytokine activation (6).

It is important to investigate any association between myxomatous mitral valve disease (MMVD) and the progression of CKD due to its high prevalence in small-breed dogs. Although the possibility of pre-renal azotemia due to heart disease rather than CKD progression should be considered, a retrospective study by Martinelli et al. (7) evaluated the prevalence of CKD and anemia in dogs with CMVD and investigated the relationships among ACVIM class, IRIS stage, and survival, observing that the prevalence of azotemia in dogs with stage B1 of the American College of Veterinary Internal Medicine (ACVIM) was 12.1%, which is higher than the prevalence of canine CKD in the United Kingdom region identified using Bayesian analysis in the previous longitudinal study (0.05–3.74%) (8). Furthermore, several studies have observed an association between the deterioration of CKD condition and severity of MMVD in dogs, including increased serum symmetric dimethylarginine concentration, progression of International Renal Interest Society (IRIS) staging, increased urine podocin, and uremia (7, 9–11). The Cardiorenal Consensus Study Group has also developed guidelines on the definition, epidemiology, pathophysiology, diagnosis, and management of CvRD in dogs and cats (12).

This study aimed to evaluate the progression of CKD in dogs with MMVD by examining the deterioration in the IRIS stage and the development of hyperphosphatemia and isosthenuria as known risk factor (13, 14) using Kaplan–Meier estimates with the log-rank test and Cox proportional hazard analysis for comparison between dogs with CKD and MMVD and dogs with CKD only.

## 2. Materials and methods

### 2.1. Case selection

This was a retrospective case–control study conducted at the Chungbuk National University Veterinary Teaching Hospital. The medical records of dogs diagnosed with CKD between May 2011 and July 2019 were retrospectively reviewed. CKD was diagnosed based on the presence of at least two episodes of minimally concentrated urine [urine specific gravity (USG) < 1.030] over a period of at least 3 months in the absence of other diseases causing polyuria or polydipsia or the absence of medications that affect USG, which include diuretics, corticosteroids, phenobarbital, and fluid therapy (15). Additional factors such as ultrasonographic changes consistent with CKD (e.g., decreased size, loss of corticomedullary definition, and/or contour irregularities of the kidneys) and the presence of renal proteinuria were also considered during diagnosis (16). The treatment of MMVD was carried out according to the ACVIM MMVD consensus statement. No drugs had been prescribed for dogs in stage B1, while in stage B2, only pimobendan (0.25 mg/kg, PO every 12 h; Vetmedin®, Boehringer Ingelheim) was prescribed. Only dogs with a follow-up interval of up to 30 days were included in this study. We excluded those with a follow-up interval period greater than 30 days at least once; those prescribed additional drugs during the follow-up period; and those receiving any drug other than pimobendan at MMVD stage B2 or any drug at stage B1. Also, dogs which died during the observation period were excluded. CKD was staged based on creatinine concentrations according to the IRIS staging guidelines as follows: stage 1 (< 1.4 mg/dL), stage 2 (1.4–2.8 mg/dL), stage 3 (2.9–5.0 mg/dL), and stage 4 (> 5.0 mg/dL). Dogs with comorbid diseases, severe hypertension (≥ 180 mmHg), and/or significant proteinuria [urinary protein to creatinine (UPC) ratio > 2.0] and those undergoing diuretic therapy were also excluded (17).

Data related to the following parameters were extracted from the medical records: body weight, body condition score (BCS), body temperature, respiratory rate, auscultation, heart rate, and systolic blood pressure measured by a Doppler. Blood samples for serum biochemistry were obtained via jugular or peripheral venipuncture, and urine for urinalysis and calculation of the UPC ratio was collected using cystocentesis. Serum biochemistry was performed using a biochemical analyzer (Hitachi 7,020, Hitachi High-Technologies Co., Tokyo, Japan); USG was measured using a refractometer; and UPC was measured using a biochemistry analyzer (Catalyst One, IDEXX Laboratories, Westbrook, ME, United States) immediately after sample collection.

### 2.2. Diagnosis of MMVD

The diagnosis of MMVD was based on clinical examination, the presence of lesions of the mitral valve apparatus (mitral leaflet thickening and/or prolapse) on 2-D echocardiography, and the presence of mitral regurgitation (MR) on color flow Doppler examination (18, 19). The cardiac size on thoracic radiographs was quantified using the vertebral heart scale (VHS) method in the right lateral view (20). An echocardiographic unit (Aloka ProSound Alpha 7, Aloka Co., Tokyo, Japan) was used to perform 2-D echocardiography, M-mode, and color flow Doppler examinations. The mitral valve structure and presence of MR were assessed using color flow Doppler examinations in the right parasternal long-axis and left apical four-chamber views, respectively (21). The left atrial-to-aortic root (LA/Ao) ratio and the left ventricular internal dimension in diastole (LVIDd) were obtained using the 2-D right parasternal short-axis view in early ventricular diastole (22, 23). The LVIDd was normalized for body weight (LVIDdN) using the following formula: LVIDd/[body weight (kg)]0.294 (24). All medical records of dogs with MMVD were re-evaluated retrospectively, and they were divided into two subgroups according to the ACVIM staging system. Stage B2 was defined with the following characteristics: murmur intensity ≥3/6, LA/Ao ≥ 1.6, LVIDdN ≥1.7, and breed-adjusted radiographic VHS > 10.5. The dogs with evidence of mild MR that did not meet these criteria were classified as stage B1 (18).

### 2.3. Criteria for CKD progression

The following criteria were used to determine CKD progression: an increase in the IRIS stage by at least one level and the development of hyperphosphatemia (serum phosphorus level > 6.2 mg/dL) or isosthenuria (USG 1.008–1.012) (17). The duration from the initial diagnosis to the IRIS stage worsening and occurrence of hyperphosphatemia or isosthenuria was evaluated.

### 2.4. Grouping

**Figure 1:**
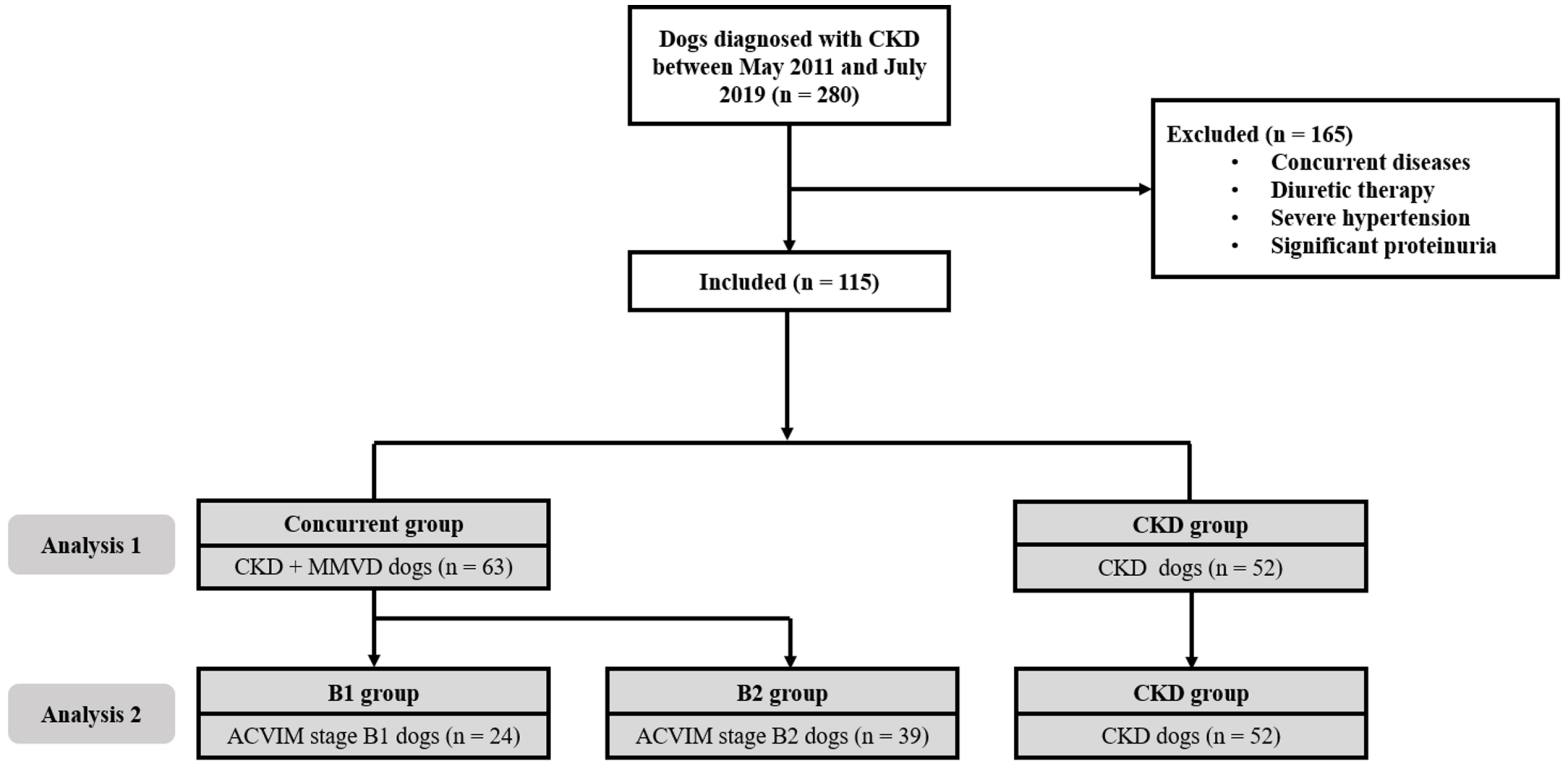
Flow diagram of the enrollment of cases in this study. In analysis 1, 63 dogs with comorbid MMVD (concurrent group) were compared with 52 dogs that had CKD alone (CKD group). In analysis 2, 24 dogs with stage B1 MMVD (B1 group), 39 dogs with stage B2 MMVD (B2 group), and 52 dogs with CKD alone (CKD group) were compared. ACVIM, American College of Veterinary Internal Medicine; CKD, chronic kidney disease; MMVD, myxomatous mitral valve disease.

We conducted two analyses. In analysis 1, the dogs with CKD were divided into two groups, i.e., dogs with MMVD (concurrent group) and dogs without MMVD (CKD group). We compared the time for CKD progression in dogs with and without comorbid MMVD (Figure 1).

In analysis 2, dogs with CKD were divided into three groups, i.e., dogs with ACVIM stage B1 MMVD (B1 group), dogs with ACVIM stage B2 MMVD (B2 group), and dogs with CKD alone (CKD group). We evaluated the time for CKD progression in these groups according to the severity of MMVD.

### 2.5. Statistical analyses

All statistical analyses were performed using commercially available statistical programs (SPSS 26.0 for Windows, IBM, Armonk, NY, United States; GraphPad Prism 8, GraphPad Software Inc., La Jolla, CA, USA). Since many values of multiplication of breed and group have an expected value lower than 5, statistical analysis was performed using the Fisher’s exact test to compare between-group differences in demographic characteristics and the distributions of the initial IRIS stages among the groups. The Shapiro–Wilk test was used to determine whether the data were normally distributed. Normally distributed data are expressed as mean and standard deviation, whereas non-normally distributed data were expressed as median and range. In analysis 1, the time for CKD progression was compared between the two groups using Kaplan–Meier estimates with the log-rank test. A Cox proportional hazard analysis was conducted to determine independent predictors of CKD progression. In analysis 2, the time for CKD progression was compared among the three groups using the analysis of Kaplan–Meier estimates with the log-rank test. Methods to handle missing data were not required because there was no missing data on the outcome in the Kaplan–Meier analysis or Cox proportional hazard analysis. *p*-values <0.05 were considered statistically significant.

## 3. Results

### 3.1. Study population

A total of 115 dogs were included in this study. Figure 1 shows the flow diagram describing the enrollment and classification of the dogs included in this study. The demographic information, including age, breed, weight, BCS, and sex of the concurrent (*n* = 63) and CKD (*n* = 52) groups is summarized in Table 1. There were no significant between-group differences in the demographic characteristics (*p* > 0.05). Most of the dog breeds included in this study were Maltese (33%) and Shih Tzu (25%).

**Table 1:** Demographic information on the concurrent and CKD groups.

	Concurrent group (*n* = 63)	CKD group (*n* = 52)	*p-*value
	CKD + MMVD	CKD alone	
Age (years)	12 (11–14)	13 (10–15)	0.47
Breeds			0.08
Maltese	25	13	
Shih Tzu	19	10	
Mixed	4	4	
Cocker spaniel	4	1	
Miniature schnauzer	3	1	
Poodle	2	4	
Yorkshire Terrier	1	7	
Spitz	1	2	
Beagle	1	0	
Pomeranian	1	1	
Pekingese	1	3	
Miniature pinscher	1	1	
Bichon Frise	0	1	
Golden retriever	0	1	
Border Collie	0	1	
Chihuahua	0	1	
Shar Pei	0	1	
Body weight (kg)	4.51 (3.22–6.35)	4.46 (2.96–6.31)	0.84
Body condition score	5 (4–5)	4 (4–5)	0.88
Sex			0.84
Female	6	7	
Neutered female	28	20	
Male	7	8	
Neutered male	22	17	
Body temperature	38.7 (38.2–39.1)	38.7 (38.3–38.9)	0.40
Heart rate	144 (120–162)	144 (120–162)	0.88
Respiratory rate	30 (24–36)	30 (24–36)	0.55
Systolic blood pressure	136 (124–150)	140 (130–160)	0.09

Age, body weight, body condition score, body temperature, heart rate, respiratory rate, and systolic blood pressure are expressed as the median (interquartile range).

CKD, chronic kidney disease; MMVD, myxomatous mitral valve disease.

The initial IRIS stages of CKD in the B1 (*n* = 24), B2 (*n* = 39), and CKD (*n* = 52) groups are shown in Table 2. There were no significant differences in the distributions of the initial IRIS stages among the three groups (*p* = 0.222). The median follow-up duration was 466 days (range: 91–1,684).

**Table 2:** Initial IRIS stage of CKD in each group.

	Concurrent (CKD + MMVD) group		CKD group
	B1 group (*n* = 24)	B2 group (*n* = 39)	CKD group (*n* = 52)
IRIS stage 1	20 (83.33%)	26 (66.67%)	25 (48.08%)
IRIS stage 2	3 (12.5%)	12 (30.77%)	24 (46.15%)
IRIS stage 3	1 (4.17%)	1 (2.56%)	3 (5.77%)
IRIS stage 4	0 (0%)	0 (0%)	0 (0%)

Categorical variables are reported as numbers (percentage).

No significant differences were found in the distributions of the initial IRIS stages among the three groups (*p* > 0.05).

CKD, chronic kidney disease; IRIS, International Renal Interest Society.

### 3.2. Comparison of the interval to IRIS stage worsening

**Figure 2:**
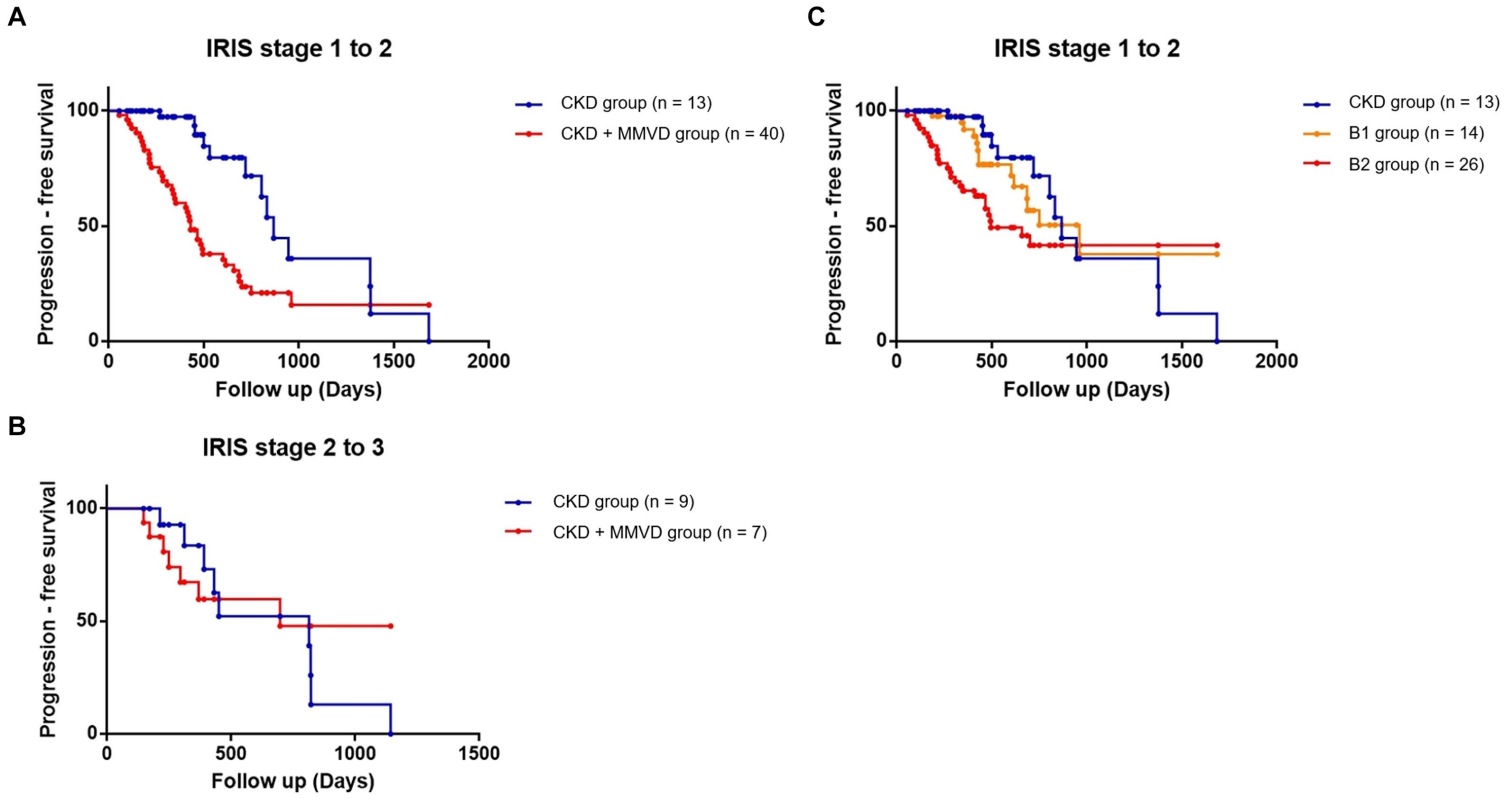
Kaplan–Meier curves comparing the time to progression from IRIS stage 1 to stage 2 between the concurrent (CKD + MMVD) and CKD groups (*p* < 0.001); **(A)** from IRIS stage 2 to stage 3 (*p* = 0.617); **(B)** and from IRIS stage 1 to stage 2 according to the severity of MMVD (*p* = 0.515); **(C)** Each dot represents an individual case.

IRIS stage worsening was observed during the follow-up period in 76.19% (*n* = 48) and 46.15% (*n* = 24) of the dogs in the concurrent and CKD groups, respectively. Of the 48 dogs of the concurrent group with progressed IRIS stage, 40 progressed from IRIS stage 1 to 2, 7 from IRIS stage 2 to 3, and 1 from IRIS stage 3 to 4. Of the 24 dogs of the CKD group with progressed IRIS stage, 13 progressed from IRIS stage 1 to 2, 9 from IRIS stage 2 to 3, and 2 from IRIS stage 3 to 4. The results of the Kaplan–Meier survival curve analysis together with the log-rank tests indicated that the time taken for worsening from IRIS stage 1 to stage 2 in the concurrent group was significantly shorter than that in the CKD group (*p* < 0.001) (Figure 2A). The results of a Cox proportional hazard analysis of factors associated with CKD progression from IRIS stage 1 to stage 2 are shown in Table 3. MMVD was associated with an increased risk of CKD progression (hazard ratio, 6.442; 95% confidence interval (CI), 2.354 to 18.850; p < 0.001). However, a significant difference was not observed between the concurrent group and the CKD group for the time taken for progression from IRIS stage 2 to 3 (*p* = 0.617) (Figure 2B). Furthermore, no significant differences were identified between the CKD, B1, and B2 groups in the multiple comparison (*p* = 0.515) (Figure 2C).

**Table 3:** Results of a Cox proportional hazard analysis of factors associated with CKD progression.

	Cases (%)	Hazard ratio	95% CI	*P*-Value
MMVD				
No	13 (25)	Reference	Reference	Reference
Yes	40 (75)	4.148	1.707–11.020	0.003^*^
Sex				
Female	30 (57)	Reference	Reference	Reference
Male	23 (43)	1.593	0.779–3.186	0.192
Body weight (kg)	53 (100)	1.044	0.978–1.104	0.144
Age (years)	53 (100)	0.974	0.856–1.111	0.685
Body condition score	53 (100)	0.888	0.632–1.228	0.480
Body temperature	53 (100)	1.434	0.645–3.157	0.372
Heart rate	53 (100)	1.004	0.999–1.018	0.576
Respiratory rate	53 (100)	0.998	0.948–1.047	0.924
Systolic blood pressure	53 (100)	0.997	0.978–1.015	0.723

MMVD, myxomatous mitral valve disease.

The asterisk indicates statistically significant difference after Cox proportional hazard analysis (*p < 0.05).

### 3.3. Comparison of the time to the occurrence of hyperphosphatemia

**Figure 3:**
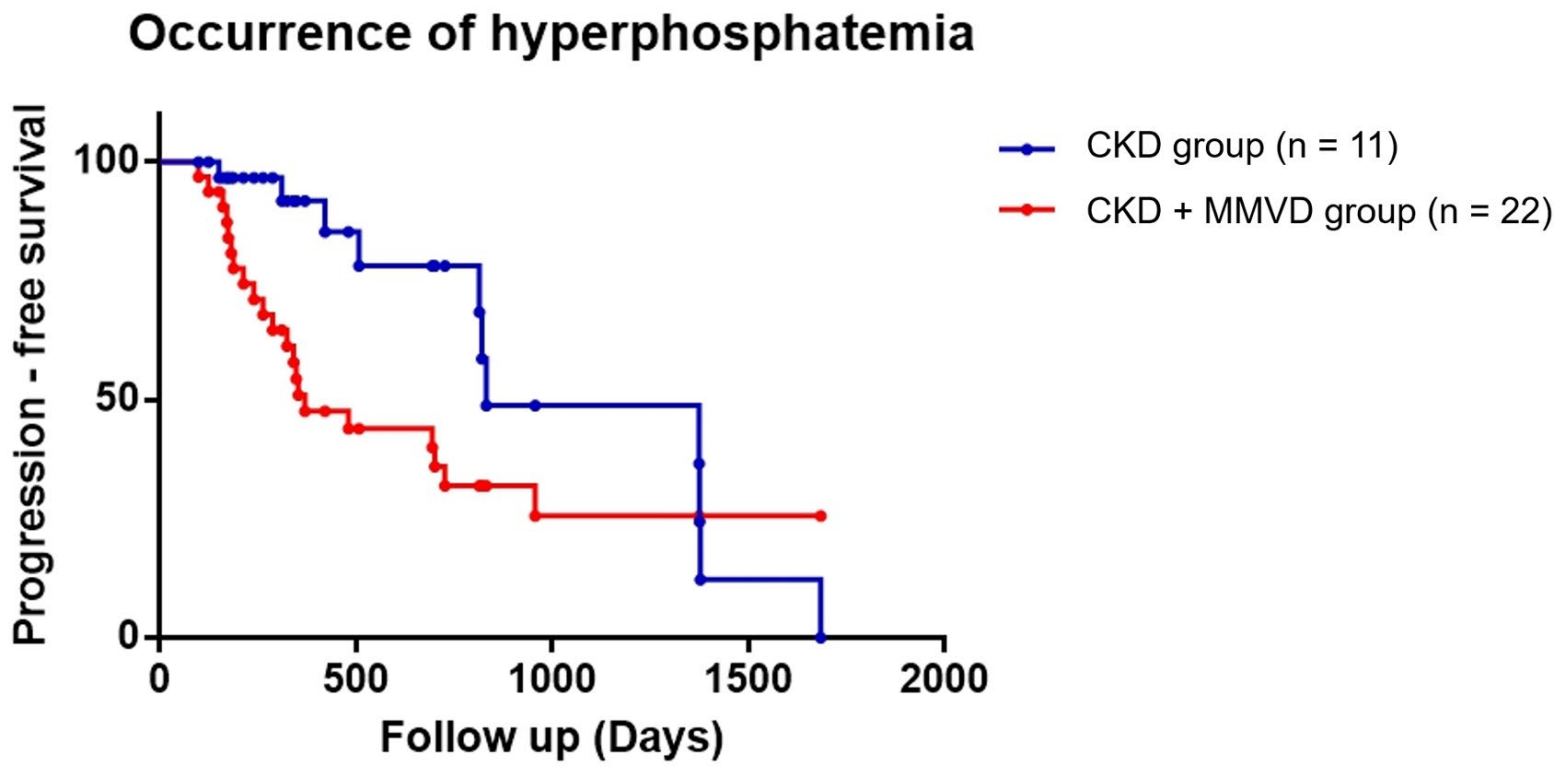
Kaplan–Meier curves of the time to the occurrence of hyperphosphatemia between the concurrent (CKD + MMVD) and CKD groups (*p* = 0.077). Each dot represents an individual case.

The data of three dogs (one in the concurrent group and two in the CKD group) with hyperphosphatemia at initial diagnosis were excluded from this analysis. The data of the remaining 112 dogs (62 in the concurrent group and 50 in the CKD group) were included in the final analyses. Hyperphosphatemia occurred in 35.48% (*n* = 22) and 22% (*n* = 11) of the dogs in the concurrent and CKD groups, respectively. Owing to the low event rate, this parameter could not be compared between B1 (*n* = 5), B2 (*n* = 16), and CKD groups. The results of the Kaplan–Meier survival curve analysis together with the log-rank tests indicated that the time for the occurrence of hyperphosphatemia in the concurrent group was not significantly shorter than that in the CKD group (*p* = 0.077) (Figure 3). Cox proportional hazards analysis could not be performed due to the low event rate.

### 3.4. Comparison of the time for the occurrence of isosthenuria

The data of 38 dogs (18 in the concurrent group and 20 in the CKD group) who were detected with isosthenuria at the initial diagnosis were excluded from this analysis. The data of the remaining 77 dogs (45 in the concurrent group and 32 in the CKD group) were included in the final analyses. Isosthenuria occurred in 48.89% (*n* = 22) and 34.37% (*n* = 11) of the dogs in the concurrent and CKD groups, respectively.

**Figure 4:**
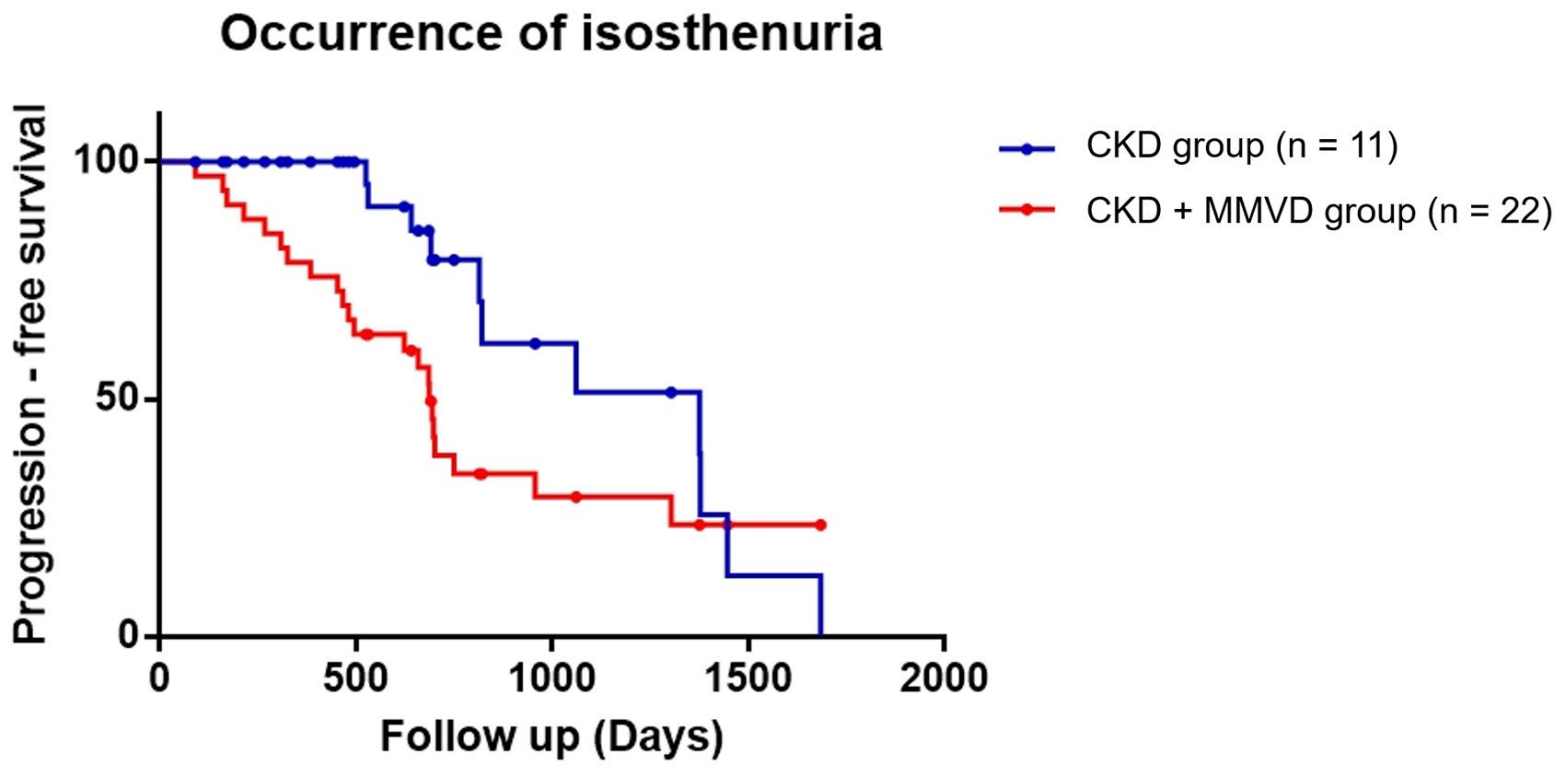
Kaplan–Meier curves of the time to the occurrence of isosthenuria between the concurrent (CKD + MMVD) and CKD groups (*p* = 0.055). Each dot represents an individual case.

The results of the Kaplan–Meier survival curve analysis together with the log-rank tests indicated that the time for the occurrence of isosthenuria in the concurrent group was not significantly shorter than that in the CKD group (*p* = 0.055) (Figure 4). Cox proportional hazards analysis could not be performed due to the low event rate.

## 4. Discussion

While we observed that CKD progressed from IRIS stage 1 to 2 faster in dogs with MMVD than in those without MMVD, there was no significant difference in the proportion of dogs developing hyperphosphatemia or isosthenuria between these two groups. In addition, MMVD was confirmed as a risk factor for CKD progression from IRIS stage 1 to IRIS stage 2 through Cox proportional hazard analysis. Although the results of all stages of CKD progression were not confirmed due to the low event rate and small sample size, our results suggest that comorbid MMVD could be considered a risk factor for the progression of CKD.

Previous studies have reported kidney dysfunction in dogs with decompensated MMVD (3, 12). However, the interactions between the renal and cardiovascular systems have not been completely elucidated. Furthermore, there is little information about the progression of renal dysfunction in dogs with comorbid MMVD. This study showed that CKD progression from stage 1 to stage 2 was observed more rapidly in CKD dogs with MMVD than in CKD alone. The presence of MMVD was associated with higher rates of CKD progression; however, its definite pathophysiology is still unclear.

The results of this study were consistent with those of previous studies in humans. The presence of cardiovascular disease was found to confer an increased risk of progression to end-stage renal disease in a cohort comprising 313 human patients with CKD (4). Moreover, a large cohort study in Taiwan reported that the incidence of CKD in human patients with heart disease was 4.1 times greater than in patients without heart disease (5). Furthermore, according to the Atherosclerosis Risk in Communities Study and the Cardiovascular Health Study, the presence of any cardiovascular disease was associated with higher rates of CKD progression (25).

Several studies have indirectly reported the existence of CvRD in veterinary medicine. A retrospective study reported that 50% of dogs with congestive heart failure (CHF) had azotemia, and its incidence increased with the severity of CHF (26). Another study revealed that serum cystatin-C and symmetric dimethylarginine concentrations were closely correlated with CHF severity (27). However, it should not be overlooked that diuretics and angiotensin-converting enzyme inhibitors used to treat CHF can cause azotemia. Diuretics induce dehydration, which leads to hypotension and hypovolemia and ultimately decreases the filtrate function of the kidney (28). Similarly, during angiotensin-converting enzyme inhibitor (ACEi) initiation, renal dysfunction can occur due to a drop in renal perfusion pressure and a subsequent decrease in glomerular filtration. This is attributed to the drug’s preferential vasodilation of the renal efferent arteriole, which impairs the kidney’s ability to compensate for low perfusion states (28, 29).

The contribution of inflammatory mediators such as tumor necrosis factor-α, interleukin-6, monocyte chemoattractant protein-1, nuclear factor kappa B, and oxidative injury in heart failure, which also leads to renal damage (30, 31). Azotemia occurred in dogs with MMVD ACVIM stage B1, suggesting that renal damage occurs even before the development of heart failure in CvRD (7). In this study, CKD progression from stage 1 to stage 2 was observed faster in CKD dogs with MMVD before the onset of CHF than in dogs with CKD alone. Therefore, inflammatory mediators associated with cardiovascular disease may be related. On the other hand, the plasma concentration of norepinephrine (NE) is not only significantly increased in dogs with CHF but also in dogs with preclinical MMVD (32). Furthermore, early sympathetic nervous system activation was detected in dogs with experimentally produced MR without any clinical signs (33). In the early stage of the disease, there is increased local sympathetic nervous system activation in the heart and kidneys (34). The NE enters the general circulation as the disease progresses, resulting in an increase in plasma NE levels and chronic sympathetic efferent neural activation, which further leads to oxidative stress (33, 35). In dogs with MMVD, the degree of increase in the circulating plasma NE levels is relative to the degree of enlargement of the heart (33, 34). Furthermore, several studies on dogs have shown that RAAS activation may be dependent on the severity and duration of cardiac disease (33, 36, 37). These findings potentially account for the relatively slow progression of CKD in dogs with stage B1 MMVD, as observed in our study. Moreover, the possibility that tissue-produced aldosterone production leads to profibrotic, proinflammatory, and hypertrophic states, leading to remodeling and dysfunction of cardiovascular and renal tissues, has been suggested through *in vitro* experiments (30).

The main limitations of this study are its retrospective design and small sample size. Second, 50% of the population were Maltese and Shih Tzu, which could potentially not reflect the more general population of breeds. Third, GFR was not measured, and this could have resulted in an erroneous estimation of renal function in some dogs. Diuretics can reduce GFR and thereby affect the levels of renal markers; hence, dogs with ACVIM stage C and D MMVD that received diuretics were not included in this study. Potential MMVD-related factors contributing to CKD, such as plasma NE, RAAS hormones, inflammatory cytokines, and oxidative stress markers, were not measured. Fourth, worsening of proteinuria and dogs with IRIS stages 3 and 4 were not examined because of the small sample size. Further studies addressing these issues are needed to provide a thorough knowledge of the complex pathophysiology of cardiovascular and kidney disease progression. Fifth, due to the retrospective nature of this study, standard treatment was not performed for all dogs. While there was no additional drug prescription during the follow-up period, we did not evaluate the existing prescribed drugs; therefore, the impact of this situation on each individual dog could not be assessed. Finally, the small number of dogs studied with hyperphosphatemia or isosthenuria may have contributed to negative findings (type 2 error).

## 5. Conclusion

The rate of progression of CKD IRIS stage 1 to stage 2 was faster in dogs with comorbid MMVD than in those with CKD alone. In addition, MMVD was confirmed as a risk factor for CKD progression from IRIS stage 1 to IRIS stage 2 through Cox proportional hazard analysis. Although no significant differences were identified for the occurrence of isosthenuria and hyperphosphatemia, since this study was conducted on a small sample, clinicians should interpret our results cautiously.

## Data availability statement

The raw data supporting the conclusions of this article will be made available by the authors, without undue reservation.

## Ethics statement

Ethical review and approval was not required for the animal study because Consent for participation in this study was obtained from the animal owners. Written informed consent was obtained from the owners for the participation of their animals in this study.

## Author contributions

HY and YK designed the research, performed the research, analyzed the data, and wrote the manuscript. YC, DL, SC, and JK analyzed the data. TY, M-PY, and HK performed the research and analyzed the data. B-TK performed the research, analyzed the data, was involved in drafting the manuscript, and will approve revisions. All authors contributed to the article and approved the submitted version.

## Funding

This work was supported by a National Research Foundation of Korea (NRF) grant funded by the Korean government (MSIT) (No. 2021R1A2C1012058) and the Basic Research Lab Program (2022R1A4A1025557) through the NRF of Korea funded by the Ministry of Science and ICT.

## Conflict of interest

The authors declare that the research was conducted in the absence of any commercial or financial relationships that could be construed as a potential conflict of interest.

## Publisher’s note

All claims expressed in this article are solely those of the authors and do not necessarily represent those of their affiliated organizations, or those of the publisher, the editors and the reviewers. Any product that may be evaluated in this article, or claim that may be made by its manufacturer, is not guaranteed or endorsed by the publisher.
